# Early-Phase Activation of Epithelial–Mesenchymal Transition in Lung Cancer Cells Treated with Epidermal Growth Factor Receptor Tyrosine Kinase Inhibitors

**DOI:** 10.3390/ijms27146207

**Published:** 2026-07-11

**Authors:** Alessia Belloni, Lorenza Tamberi, Laura Graciotti, Tatiana Spadoni, Giulia Matacchione, Chiara Giordani, Angelica Giuliani, Elisa Chiadini, Laura Capelli, Eleonora Donno, Camilla Sbrighi, Michele Zanoni, Paola Ulivi, Maria Rita Rippo, Lucio Crinò, Matteo Canale, Giuseppe Bronte

**Affiliations:** 1 Department of Clinical and Molecular Sciences (DISCLIMO), Polytechnic University of Marche, 60121 Ancona, Italy; 2 Department of Biomedical Sciences and Public Health, Polytechnic University of Marche, 60121 Ancona, Italy; 3 Clinic of Laboratory and Precision Medicine, National Institute of Health and Sciences on Ageing (IRCCS INRCA), 60127 Ancona, Italy; 4 Biosciences Laboratory, IRCCS Istituto Romagnolo per lo Studio dei Tumori (IRST) “Dino Amadori”, 47014 Meldola, Italy; 5 Department of Medical Oncology, IRCCS Istituto Romagnolo Per Lo Studio Dei Tumori (IRST) “Dino Amadori”, 47014 Meldola, Italy

**Keywords:** non-small cell lung cancer, tyrosine kinase inhibitors, epithelial-to-mesenchymal transition, gefitinib, osimertinib

## Abstract

Epithelial–mesenchymal transition (EMT) emerged as a phenotypic change associated with the resistance to epidermal growth factor receptor tyrosine kinase inhibitors, both in vitro and in vivo. The mechanisms underlying this biological process have not yet been fully understood. E-cadherin loss, N-cadherin, and vimentin increase are the main markers characterizing EMT, together with the expression of some transcription factors, such as Snail, Slug, Zeb1, Zeb2, and Twist. In this study, we explore the expression of these markers in lung cancer cell lines bearing wild-type or mutated epidermal growth factor receptor (EGFR), A549 and PC9, respectively. We treated PC9 cells with Gefitinib or Osimetinib, alone or combined with the transforming growth factor beta (TGF-β). We evaluated the expression of E-cadherin, N-cadherin, vimentin, and the transcription factors at 24 and 96 h timepoints, to verify the role of EMT in the early phases of the treatment with tyrosine kinase inhibitors (TKIs). At the 96 h timepoint, we found that in PC9 cells, the treatment with gefitinib or osimertinib, regardless of TGF-β, induces E-cadherin reduction and N-cadherin increase, similar to the effects induced by TGF-β in A549 cells at 24 h. Among the transcription factors, at 96 h, Slug mainly increases when PC9 cells are treated with gefitinib or osimertinib. These results imply that changes in the expression of these epithelial–mesenchymal transition markers may have facilitated the development of drug resistance.

## 1. Introduction

Lung cancer, similar to other malignancies, develops as a multistage process over a long period. However, the inactivation of a single oncogene can also impair cancer cell growth and survival [1]. Driver oncogene mutations usually induce a constitutively activated protein, which confers a growth advantage to the cells harboring it after clonal selection. This oncogene addiction provided the rationale for the development of targeted therapies [2]. To date, we can target a number of driver oncogene mutations by means of specific drugs found in non-small cell lung cancer (NSCLC) patients. These gene alterations include Kirsten rat sarcoma virus (KRAS) mutations, epidermal growth factor receptor (EGFR) mutations, echinoderm microtubule-associated protein-like 4-anaplastic lymphoma kinase (EML4-ALK) fusions, c-ros proto-oncogene 1 (ROS1) rearrangements, v-raf murine sarcoma viral oncogene homolog B1 (BRAF) mutations, mesenchymal–epithelial transition factor (MET) mutations, neurotrophic tropomyosin kinase receptor (NTRK) fusions, rearranged during transfection (RET) fusions, and human epidermal growth factor receptor 2 (HER2) mutations.

Among these, EGFR mutations in tissue biopsies account for 10–15% of Caucasian and around 40% of Asian NSCLC patients [3]. Previously, first- (e.g., gefitinib and erlotinib) and second-generation (e.g., afatinib) tyrosine kinase inhibitors (TKIs) were developed to treat these patients. Currently, osimertinib, a third-generation EGFR TKI, is recommended as adjuvant treatment in early-stage disease and as first-line treatment in metastatic disease of patients diagnosed with NSCLC and common mutations, i.e., exon 19 deletions (Ex19dels) and exon 21 point mutation (L858R) [4,5]. In addition, the combinations of osimertinib plus platinum-pemetrexed and amivantamab plus lazertinib recently showed higher efficacy over single-agent osimertinib [6,7]. However, like other early-generation EGFR-TKIs, osimertinib will lead to acquired resistance, limiting its effectiveness in treating patients with EGFR-mutated NSCLC. This resistance can be broadly categorized into EGFR-dependent (on-target) and EGFR-independent (off-target) types. Among the resistance mechanisms to osimertinib, the on-target secondary EGFR mutations, e.g., C797S in exon 20, are not the main observed ones. Indeed, unlike first- and second-generation EGFR-TKIs, osimertinib shows a higher incidence of acquired EGFR-independent resistance. These include MET amplifications, HER2 amplifications, phosphatidylinositol 3-kinase (PI3KCA), BRAF and RAS mutations, ALK or RET rearrangements, and cell cycle gene alterations [8,9]. The onset of these EGFR-independent resistance mechanisms during the treatment with osimertinib occurred earlier than EGFR-dependent mechanisms. We can explain this observation with the rapid rise of pre-existing subclones under the selective pressure induced by EGFR-TKIs [10]. Finally, researchers observed phenotypic changes as resistance mechanisms to EGFR-TKIs. These include the epithelial–mesenchymal transition (EMT) and the transformation of NSCLC to small-cell cancer (SCLC) [11,12].

EMT is a reversible biological program, characterized by a switch of cell phenotype from epithelial to mesenchymal state. It is involved in both normal biological processes, e.g., embryonic development, and diseases, including cancer [13]. EMT favors tumorigenesis, invasion/metastasis, and drug resistance [14]. Cells with EMT activation show a downregulation of E-cadherin, together with an upregulation of N-cadherin and vimentin [15]. Morphologically, these cells lose apico-basal polarity and cell–cell junctions, with concurrent remodeling of the cytoskeleton. These changes lead to stronger invasive capacity, drug resistance, and cancer stem-cell-like features. Since the advent of first-generation EGFR TKIs, EMT has emerged as a mechanism of drug resistance both in vitro and in vivo [16].

We aimed to verify the activation of the EMT process in the first four days of treatment with EGFR TKIs. To this end, we used two different cell lines, i.e., A549 (EGFR wild-type NSCLC cell lines) and PC9 (gefitinib sensitive cells, employed to study resistance induced by several TKIs), treated with TGF-β, to induce EMT at different short-term timepoints (24 h and 96 h). The main objective of this study, however, consisted in evaluating the expression of typical EMT markers in NSCLC cell cultures treated with EGFR-TKIs. Therefore, the experiments were designed to compare the expression of these markers: (1) in the absence of any treatment; (2) in the presence of TGF-β, capable of inducing EMT; (3) in the presence of an EGFR-TKI (gefitinib or osimertinib); and (4) in the presence of TGF-β combined with one of these EGFR-TKIs.

## 2. Results

### 2.1. Effect of Gefitinib and Osimertinib Treatment on Cell Viability

**Figure 1:**
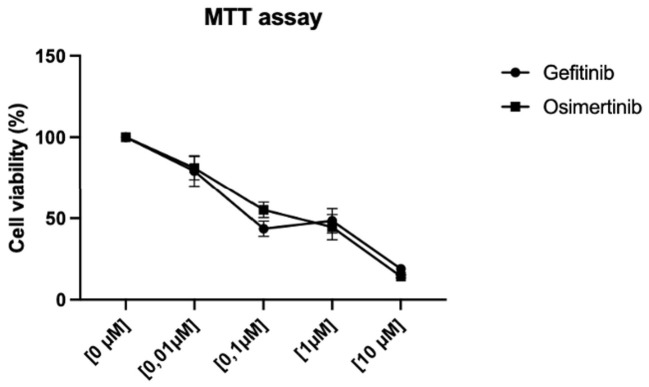
Cell viability test achieved through a MTT assay on PC9 cell line to assess gefitinib and osimertinib concentration. Results were obtained in triplicate after 96 h of treatment. *X*-axis represents drug concentrations tested, while *Y*-axis the percentage of viable cells.

To assess the concentration of EGFR-TKIs gefitinib and osimertinib for cell treatment, scalar concentrations from 0 to 10 μM of both drugs were tested on PC9 cell lines to evaluate the effect on cell viability. As reported in Figure 1, gefitinib and osimertinib exert a similar effect on cell viability, with the lower dose of the drug being less cytotoxic (0.01 μM, ~80% of cell viability); it was selected as the concentration to be employed in vitro for all the following experiments.

### 2.2. Genomic Analysis of Cancer-Associated Genes in A549 and PC9 Treated with Gefitinib or Osimertinib and TGF-β

**Figure 2:**
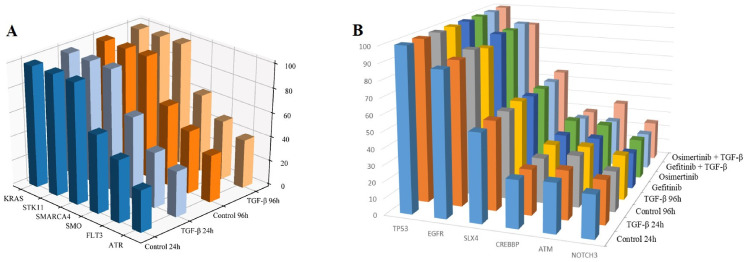
Histograms representing VAF percentage (*y*-axis) of gene mutations (*x*-axis) under each experimental condition (*z*-axis) in A549 (**A**) and PC9 (**B**) cell lines.

In order to evaluate whether different pharmacologic treatments would be able to induce genetic mutations affecting cancer-related genes, an extensive molecular characterization of cell lines under different experimental conditions was performed. The A549 cell line was treated with TGF-β for 24 h or 96 h, while for the PC9 cell line the addition of single treatment schedules with gefitinib or osimertinib and combinations of gefitinib plus TGF-β and osimertinib plus TGF-β were performed, for 96 h. A 523 gene-panel DNA/RNA assay was applied and VAF of gene mutations in treated cell lines were compared to relative non-treated ones. In both cell lines, no substantial differences were observed between treated and non-treated cells, nor in terms of appearance of new mutations or in terms of significant changes in VAF of mutations (Figure 2). For A549, mutations affecting KRAS, STK11, and SMARCA4 were always found at VAF 100%, while mutations in ATR, SMO, and FLT3 were found in VAF ranges of 35–40%, 64–68%, and 46–52%, respectively (Supplementary Table S2). For the PC9 cell line, EGFR exon 19 deletion was found at an 88–93% range, while a TP53 mutation was at VAF 100% in all experimental conditions. Other mutations were found in CREBBP (Variant Allele Frequency, VAF 28–34%), NOTCH3 (VAF 23–28%), SLX4 (VAF 54–56%), and ATM (VAF 30–33%). No gene fusions have been found in the cell lines in the 51 genes investigated.

### 2.3. Analysis of Structural EMT Biomarkers in EGFR Wild-Type and EGFR Mutated NSCLC Cell Lines After TGF-β Treatment

**Figure 3:**
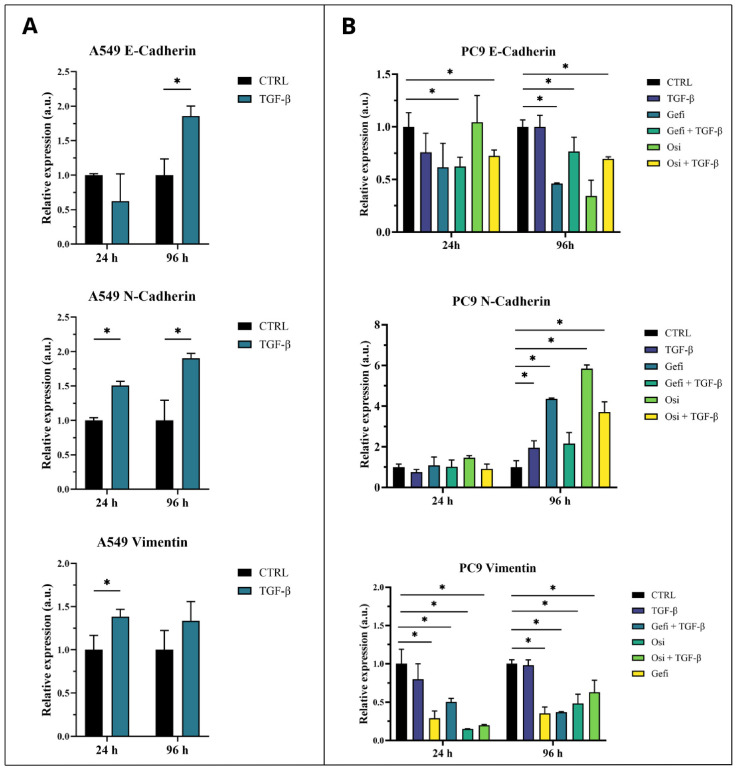
Expression of the main structural EMT biomarkers (E-cadherin, N-cadherin, and vimentin) in A549 and PC9 cell lines analysed after 24 h and 96 h of culture. (**A**) EGFR wild-type A549 cell line was treated with TGF-β, and E-cadherin, N-cadherin and vimentin mRNA expression were evaluated after 24 h and 96 h of treatment. (**B**) EGFR-mutated PC9 cell line was treated with TGF-β, EGFR-TKIs, and combinations, and E-cadherin, N-cadherin and vimentin mRNA expression were evaluated after 24 h and 96 h of treatment. Histograms represent the mean of three independent experiments ± SD. Paired *t* test was performed for each marker with respect to the control ones; Holm–Šidák correction was performed to avoid false positives; asterisks mean statistically significant values (* *p* < 0.05). Relative gene expression values (2^−ΔCt^) were normalized to the corresponding control samples, which were assigned a value of 1. CTRL: Control; Gefi: gefitinib; Osi: osimertinib.

The effect of TGF-β treatment (24 and 96 h) on the mRNA expression of the main proteins involved in EMT, E-cadherin, N-cadherin, and vimentin was analysed in EGFR wild-type (A549) and EGFR mutated (PC9) cells by RT-qPCR analysis. The expression of E-cadherin in A549 cells demonstrated the highest values after 96 h of culture, with unexpectedly higher levels in those treated with TGF-β compared to control cells (CTRL) (Figure 3A). Conversely, the TGF-β demonstrated to induce EMT in N-cadherin and in vimentin expression: even if it was higher after 96 h than after 24 h in control samples, in both timepoints, TGF-β induced a significantly stronger expression of mRNA compared to the relative control ones. As for vimentin, TGF-β recorded the highest values at the 24 h timepoint. A different behavior was observed in the PC9 cell line (Figure 3B): both E-cadherin and vimentin expressions were slightly decreased after 24 h of TGF-β treatment, but increased again after 96 h to values similar to untreated cells, whereas N-cadherin showed a significant increase at 96 h in TGF-β treated cells. As opposed to what was achieved in the EGFR wild-type cells, the EGFR mutated ones revealed a decreasing trend in vimentin expression with values similar to control at 96 h of TGF-β exposure. 

### 2.4. Evaluation of Structural EMT Biomarkers in EGFR Mutated Cells After Blocking EGFR Receptor Through EGFR-TKIs

Similarly, RT-qPCR analysis of the expression of E-cadherin, N-cadherin, and vimentin before and after TGF-β treatment was performed on the PC9 cell line after the administration of a first-generation and a third-generation EGFR-TKI, gefitinib and osimertinib, respectively, and after the combination of one of these drugs with TGF-β. The most significant results seemed to be those observed in the reduction of E-cadherin expression after 96 h both after gefitinib and osimertinib administrationcompared to the control group, and in the expression of N-cadherin, which significantly increased after 96 h in gefitinib-treated PC9 and to a greater extent in osimertinib-treated ones. Conversely, both EGFR-TKIs decreased the vimentin expression compared to the control group. The combination of gefitinib with TGF-β and osimertinib with TGF-β also revealed significant results: E-cadherin expression was significantly reduced at both 24 h and 96 h compared with the respective control groups, with a greater decrease observed after 24 h. However, after 96 h, E-cadherin expression remained higher than that observed in cells treated with the EGFR-TKI alone. Similar results at the latest time point were observed for N-cadherin, where the combination with TGF-β decreased the level of the protein compared to the single EGFR-TKI treatment, but with higher values than the relative controls. Vimentin expression in combination treatments remained lower compared to controls, and seems not to be so different following the addition of TGF-β in gefitinib treated cells after 96 h, while a slight increase was achieved in osimertinib + TGF-β, which in any case remained below the values of the control group (Figure 3B).

### 2.5. Immunofluorescence to Assess the In-Vitro Expression of E-Cadherin and Vimentin

**Figure 4:**
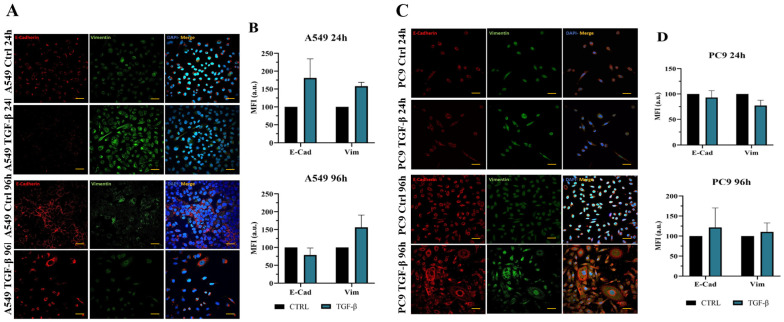
Representative confocal immunofluorescence images of E-cadherin (red), vimentin (green), and nuclei (DAPI, blue) in A549 cells cultured under control conditions or treated with TGF-β for 24 and 96 h. (**A**) Immunofluorescence of markers in A549 cell line treated with TGF-β for 24 h and 96 h; (**B**) quantitative analysis of E-cadherin and vimentin fluorescence intensity in A549 cells treated with TGF-β for 24 h and 96 h; (**C**) Immunofluorescence of markers in PC9 cell line treated with TGF-β for 24 h and 96 h; (**D**) quantitative analysis of E-cadherin and vimentin fluorescence intensity in PC9 cells treated with TGF-β for 24 h and 96 h. Scale bar = 20 μm.

The expression of E-cadherin and vimentin was analysed by immunofluorescence, revealing interesting expression of their two EMT structural biomarkers. The choice to assess only these two was related to the higher expression of E-cadherin and vimentin with respect to N-cadherin, which revealed very low levels of mRNA. In accordance with RT-qPCR results, A549 cells showed higher E-cadherin expression in control samples than TGF-β-treated ones, with an increase at 96 h compared to 24 h in both groups (Figure 4). Through quantitative analysis on immunofluorescence images, an increase of E-cadherin was observed in the A549 cell line after 24 h of treatment, which was not observed after 96 h; on the other hand, vimentin was expressed more after 96 h of treatment. In the PC9 cell line, there was no substantial difference between the two markers and control after 24 h and 96 h of treatment with TGF-β, even though a peculiar cellular phenotype, with elongated and spindle-shaped cells, was observed (Figure 4C).

**Figure 5:**
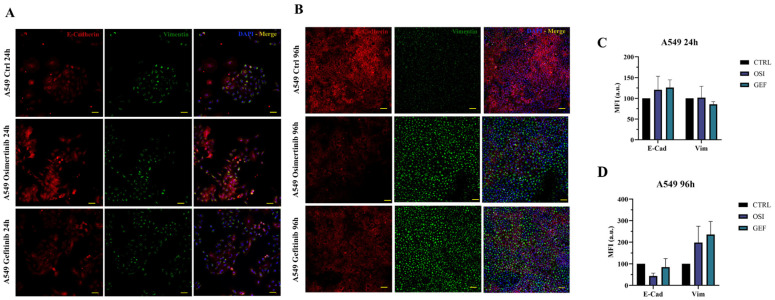
Representative confocal immunofluorescence images of E-cadherin (red), vimentin (green), and nuclei (DAPI, blue) in A549 cells cultured under control conditions or treated with tyrosine kinase inhibitors. (**A**) Immunofluorescence of markers in A549 cell line control and after 24 h treatment with osimertinib or gefitinib; (**B**) Immunofluorescence of markers in A549 cell line control and after 96 h treatment with osimertinib or gefitinib; (**C**) quantitative analysis of E-cadherin and vimentin fluorescence intensity in A549 cells treated with osimertinib or gefitinib for 24 h, with respect to control; (**D**) quantitative analysis of E-cadherin and vimentin fluorescence intensity in A549 cells treated with osimertinib or gefitinib for 96 h, with respect to control. Scale bar = 20 μm.

Then, we treated the two cell lines with TKIs to find differences in E-cadherin and vimentin protein expression. In the A549 cell line, osimertinib and gefitinib did not lead to substantial changes in terms of expression of the two markers investigated after 24 h of treatment (Figure 5A). This was also confirmed by quantitative analysis of immunofluorescence (Figure 5C). Interestingly, even though the A549 cell line carries wild-type EGFR, treatment with the two TKIs for 96 h led to a significant increase of vimentin protein expression, and a concomitant decrease in E-cadherin protein expression (Figure 5B,D). Together with the results achieved in RT-qPCR, this suggest that EGFR blockade could lead to EMT-related markers even in wild-type EGFR cells.

**Figure 6:**
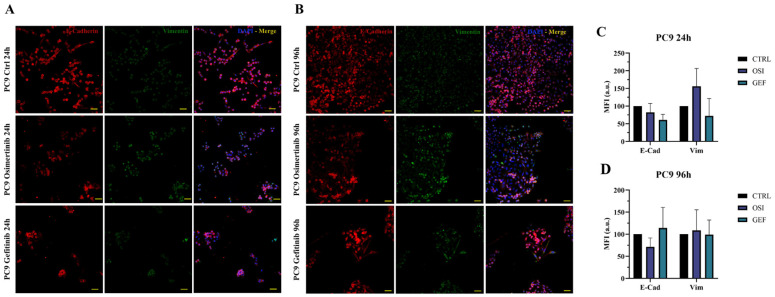
Representative confocal immunofluorescence images of E-cadherin (red), vimentin (green), and nuclei (DAPI, blue) in PC9 cells cultured under control conditions or treated with tyrosine kinase inhibitors. (**A**) Immunofluorescence of markers in PC9 cell line control and after 24 h treatment with osimertinib or gefitinib; (**B**) Immunofluorescence of markers in PC9 cell line control and after 96 h treatment with osimertinib or gefitinib; (**C**) quantitative analysis of E-cadherin and vimentin fluorescence intensity in PC9 cells treated with osimertinib or gefitinib for 24 h, with respect to control; (**D**) quantitative analysis of E-cadherin and vimentin fluorescence intensity in PC9 cells treated with osimertinib or gefitinib for 96 h, with respect to control. Scale bar = 20 μm.

In the PC9 cell line, treatment with the TKIs led to a different behavior over time (Figure 6). Interestingly, after 24 h of treatment, both osimertinib and gefitinib led to a decrease in E-cadherin expression, with a concurrent increase of vimentin for Osimertinib treated cells, but not for gefitinib treated cells (Figure 6C). At 96 h, there was no substantial difference in the protein expression of the two markers, independently of the treatment performed. These data suggest that osimertinib and gefitinib are able to induce EMT marker expression in the very early phases of treatment in the EGFR-mutated cells.

### 2.6. Evaluation of EMT-TFs mRNA Expression in EGFR Wild-Type and Mutated Cells

**Figure 7:**
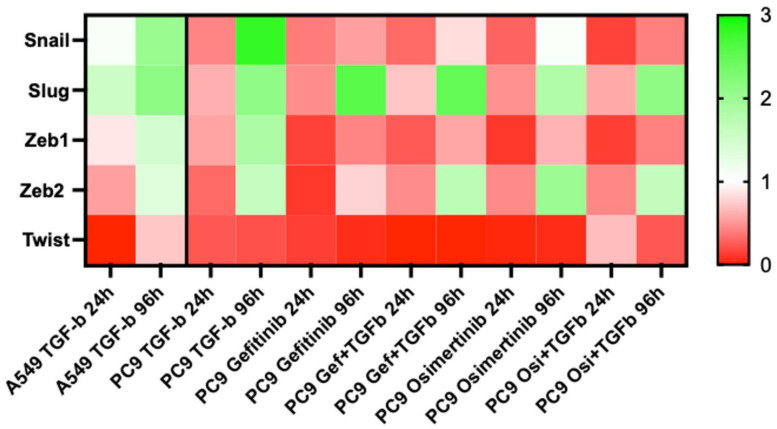
Heatmap representing trends of EMT-TFs values obtained from A549 and PC9 cell lines analysed after 24 h and 96 h. Data are presented as X-fold values compared to control samples at the same time point. Color scale represents the values corresponding to 1 in white, values up to zero in red, and values greater than 1 in green. Results are reported as the mean of three independent experiments.

To assess the trend of the main transcription factors related to EMT, Snail (Snai1), Slug (Snai2), Zeb1, Zeb2, and Twist, their evaluation was performed in A549 EGFR wild-type cells and in EGFR mutated PC9 (Figure 7). In the latter, the analysis was performed after TGF-β treatment, but also after the employment of both EGFR-TKIs, gefitinib and osimertinib, both used alone and in combination with TGF-β. The heatmap representation clearly highlights the upregulation of all transcription factors in A549 cells after TGF-β administration at 24 h and to a greater extent at 96 h, with the exception of Twist. In PC9 cells, TGF-β increased the level of Snail, Slug, and Zeb1 at 96 h. Snail reported a profile similar to Zeb1, where EMT induction with the cytokine registered the highest values, followed by the values obtained by the osimertinib and TGF-β combination. Moreover, Slug represents the most upregulated factor, especially after the administration of TGF-β and gefitinib, and to a lesser extent after osimertinib and its combination with TGF-β at 96 h. As for the less expressed Zeb2 and Twist, what emerged were the highest values of Zeb2 recorded at 96 h, especially in osimertinib treated cells and in those treated with its combination with TGF-β. Twist, instead, demonstrated downregulation in all treatments, with the exception of the combination of osimertinib and TGF-β at 24 h. Statistical analysis performed on this data is reported in Table S3.

### 2.7. Wound-Healing

**Figure 8:**
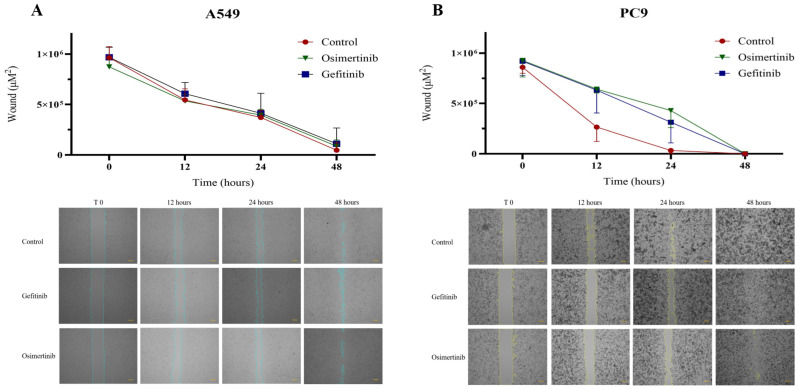
Cell migration assay of A549 (**A**) and PC9 (**B**) treated with gefitinib or osimertinib with respect to control. At the top of each panel, quantitative analysis of wound closure is shown, while at the bottom of each panel 4× magnification (scalebar 20 μM) is reported, at experimental time-points of every 12 h until wound closure. Software automatically derived area included for analysis is marked.

To further investigate whether the molecular changes observed following TGF-β and EGFR-TKI exposure were associated with functional EMT-related features, cell migration was assessed using a wound-healing assay. As shown in Figure 8, 549 cell line growth was slightly affected by EGFR-TKIs (Figure 8A). Interestingly, in the PC9 cell line, treatment induced changes in the migratory behavior of cells compared with the corresponding untreated control (Figure 8B). In particular, quantitative analysis of wound closure demonstrated that PC9 cell growth was affected during the first hours of treatment (T0—24 h) while after 48 h of treatment with TKIs, cells showed enhanced migration and closed the wound at the control level, supporting the acquisition of EMT-associated phenotypic traits (Figure 8B). Upon visual inspection, cells indeed showed an EMT-related shape, demonstrating early changes in cellular phenotype. Even though the drug was administrated at a sub-lethal dose, these findings are consistent with the transcriptional and immunofluorescence data, suggesting that EGFR-TKIs exposure promote early adaptive changes associated with EMT.

## 3. Discussion

In this study, we focused on EMT markers in NSCLC cell lines, A549 as EGFR wild-type cells, and PC9 as EGFR-mutated cells. We evaluated the genomic profile of these cells to verify the EGFR mutation status, but also to identify any mutations in other genes. We used an NGS method with the panel that is usually applied to the study of tumor tissue samples to focus attention on those driver genes that may have clinical relevance, especially for the identification of mutations associated with resistance to EGFR-TKIs. The analysis of the cell lines used in this study confirmed the presence of exon 19 deletion in PC9 cells, and the absence of any EGFR mutations in A549 cells. Furthermore, the overall mutational profile in these two cell lines was completely different. Specifically, A549 cells presented a G12S mutation of the KRAS gene, associated with a mutation of STK11 gene of certain pathogenetic significance, which also likely represents driver mutations in relation to the VAF. On the contrary, in addition to the aforementioned EGFR mutation, PC9 cells present with a VAF of around 90%, as well as a mutation of the TP53 gene, R248Q, affecting exon 7, with a VAF of 100%. This observation could suggest that in the context of PC9 cells, some cells could have the TP53 mutation, but not the EGFR one. The presence of TP53 mutations could underlie the development of primary resistance mechanisms to EGFR-TKIs [17,18,19]. Furthermore, genomic analysis showed that the allelic frequency does not change in relation to treatment with TGF-β and/or EGFR-TKI, and that no difference is observed in VAF between 24 and 96 h. Clearly, a longer treatment time is needed to observe a clonal selection of mutations that could modify the VAF [20]. These findings support that EMT-associated changes occur in the absence of detectable genomic evolution, consistent with a non-genetic adaptive response to EGFR-TKI exposure.

According to the main objective of this study, the EGFR-TKI treatment was performed only on PC9, as they are EGFR-mutated, to see the effect of the EGFR mutation or its inhibition on the expression of EMT markers over 96 h. As EMT markers, we considered molecules that we defined as structural markers. E-cadherin and N-cadherin, in fact, have a role in the interactions between cells; vimentin, instead, has a role in the cytoskeletal structure [21,22]. In wild-type EGFR cells at 24 h, a non-significant reduction of E-cadherin is observed together with an increase in N-cadherin and vimentin, following stimulation with TGF-β compared to the control. However, at 96 h, all three markers are more expressed both in the control and under TGF-β stimulation, a possible consequence of the greater confluence of the cells at this timepoint. In cells with EGFR mutation, stimulation with TGF-β does not determine a significant variation of these three markers compared to the control, either at 24 or 96 h. This may suggest that the EGFR mutation could interfere with TGF-β-mediated EMT, maintaining the cells with an epithelial phenotype. In fact, EGFR blockade by EGFR-TKIs, administered together with TGF-β, seems to determine a significant reduction of E-cadherin both at 24 and 96 h in comparison with the control, as well as an increase in N-cadherin at 96 h with the same type of treatment, TGF-β + EGFR-TKI. Interestingly, this effect appears more evident with osimertinib than with gefitinib. Instead, an increase in vimentin was not observed, as one might expect, perhaps because this marker would require a longer treatment duration to show an increase.

The expression of E-cadherin and vimentin as proteins was evaluated by immunofluorescence, thus providing an imaging approach able to assess the expression of the more expressed structural markers and then of their localization among the cells. This evaluation highlighted that in wild-type EGFR cells at 24 h, stimulation with TGF-β induced the loss of E-cadherin expression and a greater expression of vimentin, although this effect was lost with more prolonged stimulation at 96 h. This observation is consistent with what was observed by RT-qPCR. Interestingly, this phenomenon is also observed in A549 cell lines that have developed resistance to gefitinib, compared to sensitive ones [23]. Using treatment with TKIs, the two cell lines showed different behavior in terms of protein expression of E-cadherin and vimentin. In the A549 cell line, EMT activation was more pronounced after 24 h of treatment, while in the PC9 cell line, an increase of vimentin with a concurrent decrease in E-cadherin expression was observed after 24 h of treatment, while these differences were lost after 96 h of treatment with TKIs.

In parallel, the transcription factors associated with EMT were evaluated. Among these, the best known are Snail, Slug, Zeb1, Zeb2, and Twist [24]. Their transcriptomic analysis shows that stimulation with TGF-β requires 96 h to lead to an increase in their expression compared to the control in both cell types. Instead, in the case of treatment of PC9 with EGFR-TKI alone or associated with TGF-β, Slug is predominantly increased at 96 h, and Zeb2 partly increased. This observation agrees with data from other studies, which show that the expression of Slug, but not that of the other EMT-TFs, in particular Snail, Twist, and Zeb1, is increased in PC9 cells treated with gefitinib [25]. This phenomenon makes these cells resistant to apoptosis induced by gefitinib. The suppression of Slug can make the cells sensitive to gefitinib again [26,27]. In our study, the overexpression of Slug, but not of Snail, Twist, and Zeb1, is also observed for osimertinib, and in the combined treatment of these EGFR-TKIs with TGF-β. Through this analysis it is possible to know that treatment with EGFR-TKIs induces an increase in Slug and Zeb2 after 96 h of treatment. This probably lays the foundation for resistance to these drugs in the first four days of treatment, with mechanisms not fully related to EMT, since other EMT-TFs such as Snail, Twist, and Zeb1, are not increased. A study also shows that Slug promotes the invasiveness of tumor cells through the increase in metalloproteinase-2 activity and the suppression of E-cadherin [25]. Accordingly, in our study, the expression of Slug was also increased in the same treatment conditions, in which E-cadherin was significantly reduced compared to the control (TKI or TKI + TGF-β at 96 h). Another study has highlighted how the Slug protein can be stabilized in the presence of the TP53 mutation [28,29]. Although in our study we did not evaluate the protein expression of Slug; the PC9 cells that were studied presented 100% VAF for TP53 mutation. This would imply that the induction of Slug expression in cells treated with EGFR-TKIs and with TP53 mutation would favor greater aggressiveness and resistance to these drugs.

In the cell migration assay, while wild-type A549 cell line growth was not affected by TKI treatment, we observed that PC9 cell growth was affected during the first hours of treatment; interestingly, the results obtained in protein expression assessment by immunofluorescence showed that in the PC9 cell line, EMT marker expression was more prominent after 24 h of treatment compared to 96 h, suggesting a very early activation of the EMT program in response to treatment.

We acknowledge the limitations of the present study: the use of long-term established cell lines could lead to experimental artifacts, but these models remain essential for dissecting fundamental and conserved mechanisms of drug response. In this study, A549 and PC9 cells enabled a controlled, mechanism-focused analysis of EMT-associated plasticity in EGFR-mutant versus wild-type contexts, minimizing the confounding variability inherent to patient-derived systems. Moreover, the choice of different generations of TKIs could lead to differences in data interpretation; in this study, the inclusion of gefitinib and osimertinib was intended as a mechanistic approach to interrogate whether EGFR inhibition, independently of drug generation or clinical context, is sufficient to trigger early EMT-associated plasticity. Although our data consistently support EMT-associated molecular and functional changes induced by EGFR-TKIs, we acknowledge that additional analyses, including detailed morphological quantification and EMT transcription factor nuclear localization (such as Twist), were not included and represent important directions for future mechanistic investigation.

## 4. Materials and Methods

### 4.1. Experimental Design

**Figure 9:**
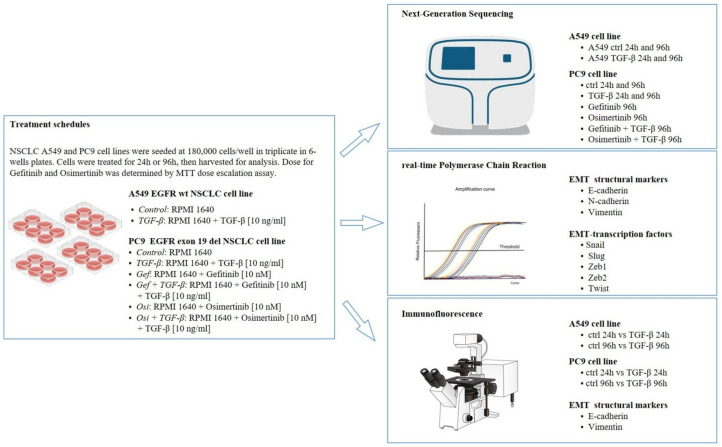
Study design summarizing the experimental setup and the analyses performed.

A549 (ATCC—Rockville, MD, USA, catalog number CCL-185) and PC9 (Cell Line Service GmbH, Eppelheim, Germany, CLS catalog number 305045) cell lines, used as control and as EGFR-mutated models, respectively, were cultured with Roosevelt Park Memorial Institute culture medium (RPMI 1640) supplemented with 10% Fetal Bovine Serum, 1% Penicillin/Streptomycin, and 1% L-Glutamine (all from Euroclone, Milano, Italy) and maintained in humidified atmosphere at 37 °C and 5% CO_2_. All cell lines were maintained both with RPMI 1640 alone (control) and with Human Transforming Growth Factor- β1 (TGF-β1) (Peprotech, Cranbury, NJ, USA; Cat. No 100-21) (TGF-β) directly added to the RPMI 1640 at a final concentration of 10 ng/mL, in accordance with the literature [30]. According to the results obtained by the MTT assay, PC9 cells were treated with 0.01 μM of gefitinib and 0.01 μM of osimertinib, directly added to the culture medium after reconstitution, as recommended, and respectively named Gef (gefitinib) and Osi (osimertinib). A cytotoxicity assay was not performed on EGFR wt A549 cells, as this cell line harbors wild-type EGFR and is widely recognized as intrinsically less sensitive to EGFR-TKIs at clinically relevant concentrations. Therefore, gefitinib and osimertinib doses established for the PC9 cell line were applied to A549 cell line, to evaluate EMT activation under drug exposure experimental conditions. Additional PC9 experimental groups named Gef + TGF-β (0.01 μM gefitinib + 10 ng/mL TGF-β) and Osi+ TGF-β (0.01 μM gefitinib + 10 ng/mL TGF-β) were cultured and studied. The medium was properly changed every 48 h and cells were trypsinised (Trypsin from Corning) when approximately 80% confluent. All cell lines were routinely tested for mycoplasma. Each A549 and PC9 experimental group was seeded in triplicate in six-well plates (Corning Costar, Sigma Aldrich, St. Louis, MO, USA) with a density of 180,000 cells/well, thus reaching the optimal confluence density at the last time point considered. Cells were collected after 24 h and after 96 h after seeding, and cell pellets containing about 200,000–800,000 cells, respectively, for 24 h and 96 h samples, were harvested after trypsinization, centrifuged at 1000 rpm × 5 min, and stored at −80 °C until DNA and mRNA extraction. A schematic representation of the study design has been reported in Figure 9.

### 4.2. Cell Viability Assay

An MTT (3-(4,5-dimethylthiazol-2-yl)-2,5-diphenyltetrazolium bromide) assay was performed to assess PC9 cell viability after gefitinib (ZD1839—Selleckchem; Cat. No S1025) and osimertinib (AZD9291—Selleckchem; Cat. No S7297) treatments. Cells were cultured in 96-well plates for 72 h (3 × 103 cells/well) and then treated with five different doses (0.01 μM, 0.1 μM, 1 μM, and 10 μM) of each EGFR-TKI (starting from 1 mM concentration of each solution). A total of 10 µL/100 µL medium of MTT solution (5 mg/mL) was added in each well and incubated for 3 h. Insoluble formazan salts were solubilized by adding 400 µL of DMSO. The absorbance was measured at OD 540 nm using a microplate reader (NB-12-0035 Microplate Reader, NeoBiotech Co., Seoul, Republic of Korea). Data are expressed as a percentage, according to the equation (T/C) × 100%, where T and C represent the mean OD of treated cells and untreated cells (control group), respectively.

### 4.3. Analysis of Cancer-Associated Genes by Next Generation Sequencing

Cells were thawed on ice and centrifuged at 150× *g* for 5 min. DNA and RNA were purified using the AllPrep^®^ DNA/RNA Mini Kit (Qiagen, Hilden, Germany), following manufacturer instructions. DNA and RNA quantity and quality were assessed by Qubit dsDNA BR Assay Kit on a Qubit fluorometer (ThermoFisher Scientific, Waltham, MA, USA). Next-Generation Sequencing (NGS) analysis was performed by the OncomineTM Comprehensive Assay v3 (OCA) v3 panel (ThermoFisher Scientific, Waltham, MA, USA). This amplicon-based DNA/RNA assay investigates alterations in 161 cancer-associated genes: in particular, hotspot mutations in 87 genes, focal CNV gains in 43 genes, full CDS for DEL mutations in 48 genes, and 51 gene fusion drivers. DNA and RNA libraries were generated starting with 10 ng of DNA and 10 ng of RNA, respectively; RNA libraries were generated by complementary DNA (cDNA), synthetized by RNA using SuperScript VILOTM Master Mix (ThermoFisher Scientific, Waltham, MA, USA). Libraries were generated using the OCA v3 DNA/RNA Chef-Ready kit, following manufacturer instructions. on an Ion Chef System (ThermoFisher Scientific, Waltham, MA, USA); then, libraries were pooled and loaded onto the Ion ChefTM System for template preparation using the Ion 540TM Kit-Chef, and sequenced on an Ion S5 Plus platform using the Ion 540 Chips (ThermoFisher Scientific, Waltham, MA, USA).

Quality control on sequencing reads, including chip loading density, median read length, and number of mapped reads, was carried out using Torrent Suite Server. Then, Ion ReporterTM software v.5.22 was used for variant calling, filtering, and annotations. Variants with a Variant Allele Frequency (VAF) equal to or above 5% and with coverage > 500× were considered. Gene alterations were called using human genome 19 (hg19) as a reference. Variants were classified using the Association for Molecular Pathology, American Society of Clinical Oncology, and College of American Pathologists Consensus classifications, defined as “tiers of actionability” [31,32].

### 4.4. RNA Isolation and mRNA Expression by RT-qPCR

Relative mRNA expression was calculated using the 2^−ΔCt^ method, normalizing each target gene to the corresponding housekeeping gene. For each experimental condition, three independent biological replicates were performed. Within each biological replicate, technical triplicates were included to ensure measurement reliability, and mean Ct values were used for subsequent calculations. For graphical representation, fold-change values were calculated relative to the control condition. Total RNA was isolated using the Total RNA Purification kit (Norgen Biotek Corp., #37500, Thorold, ON, Canada), following the manufacturer’s instructions. Total RNA quantification was determined by spectrophotometric quantification with Nanodrop ONE (NanoDrop Technologies, Wilmington, DE, USA) and reverse transcribed using PrimeScriptTM RT Reagent Kit with gDNA eraser (Cat. #RR047A, Takara), following the manufacturer’s instructions. mRNA expression was evaluated by RT-qPCR using TB GreenTM Premix Ex TaqTM (Cat#RR420A, Takara, Saint-Germain-en-Laye, France) in a Rotor-Gene Q (Qiagen). The following primers were all acquired from Sial (S.i.a.l. srl Roma, Italy): E-cadherin F: 5′-CGAGAGCTACACGTTCACGG-3′; E-cadherin R: 5′-GGGTGTCGAGGGAAAAATAGG-3′; N-cadherin F: 5′-ACAGTGGCCACCTACAAAGG-3′; N-cadherin R: 5′-CCGAGATGGGGTTGATAATG-3′; vimentin F: 5′- GAGAACTTTGCCGTTGAAGC-3′; vimentin R: 5′-GCTTCCTGTAGGTGGCAATC-3′; Twist F: 5′-AAGGCATCACTATGGACTTTCTCT-3′; Twist R: 5′-GCCAGTTTGATCCCAGTATTTT-3′; Snail (Snai1) F: 5′-CCCCAATCGGAAGCCTAACT-3′; Snail (Snai1) R: 5′-GCTGGAAGGTAAACTCTGGATTAGA-3′; Slug (Snai2) F: 5′-TGGTTGCTTCAAGGACACAT-3′; Slug (Snai2) R: 5′-GTTGCAGTGAGGGCAAGAA-3′; Zeb-1 F: 5′-GCACCTGAAGAGGACCAGAG-3′; Zeb-1 R: 5′-TGCATCTGGTGTTCCATTTT-3′; Zeb-2 F: 5′-AAGCCAGGGACAGATCAGC-3′; Zeb-2 R: 5′-CCACACTCTGTGCATTTGAACT-3′; β-actin F: 5′-TGCTATCCCTGTACGCCTCT-3′; and β-actin R: 5′-GTGGTGGTGAAGCTGTAGCC-3′. All primers were purchased as validated by the manufacturer, and were used following manufacturer annealing protocols. Data are shown both as relative expression (EMT structural markers) according to the 2^−ΔCt^ method and as fold change vs. control samples (EMT-TFs markers) at the relative time point, by using β-actin as the housekeeper in both cases. The use of β-actin as a single housekeeper was determined after checking the stability expression of the gene (Supplementary Table S1).

### 4.5. Immunofluorescence

A549 and PC9 cells were cultured in the presence or absence of TGF-β, osimertinib or gefitinib, at 24 and 96 h. The 24-h time point was chosen to capture early transcriptional and signaling events following TGF-β and EGFR-TKI exposure, whereas the 96-hour time point was selected to evaluate the persistence and stabilization of EMT-associated changes over time. Then, cells were fixed with 4% paraformaldehyde, permeabilized with 0.1% Triton X100, and incubated with 5% BSA. Primary antibodies were incubated overnight at 4 °C, while secondary antibodies were incubated for 45 min at room temperature. Nuclei were stained with DAPI or Hoechst 33342. Anti-E cadherin (Cat. AC-A86102-10) and vimentin (Cat. AC-A85420-10) antibodies were purchased by Sial (Rome, Italy). Images were acquired with an inverted microscope Eclipse Ti2-E supplied with an AX confocal system (Nikon Corporation, Japan) at 20× magnification, equipped with a Plan Apo 20×. All images were processed using NIS Elements software, v. 7.01.00. Quantitative analysis on immunofluorescence images was performed using ImageJ software (v.1.54t). Mean Fluorescence Intensity (MFI) was calculated on selected Regions of Interest (ROIs) of three different images at 4× magnification per condition, after background subtraction.

### 4.6. Wound-Healing Assay

The effect of gefitinib and osimertinib on PC9 and A549 cell migration was evaluated using a scratch migration assay. Briefly, PC9 or A549 cells were seeded into the two reservoirs of the Culture-Insert at a density of 4 × 10^5^ cells/well and maintained under standard culture conditions for 24 h until the appropriate cell confluence was reached.

Culture-Insert was then gently removed to generate a defined cell-free gap between the two cell monolayers and both cell lines were treated with 0.01 nM of osimertinib or 0.01 nM gefitinib. Images were acquired using a Nikon Eclipse Ti2 microscope equipped with a Plan Fluo 4×, 0.13 NA objective, and a Nikon DS-Fi3 digital camera (1/1.8-inch color CMOS image sensor; sensor size: 6.91 × 4.92 mm; square pixels of approximately 2.4 µm side length; 2880 × 2048 pixel resolution) (Nikon Corporation, Tokyo, Japan). Images were acquired immediately after removal of the Culture-Insert, corresponding to 0 h, and again at 12, 24, and 48 h. The wound/gap area was quantified by densitometric analysis using ImageJ software v.1.54t (U.S. National Institutes of Health, Bethesda, MD, USA). Wound closure was calculated according to the following formula: wound closure (%) = (A_0_ − A_n_)/A_0_ × 100 where A_0_ represents the initial gap area at 0 h, and A_n_ represents the residual gap area at the indicated time point, either 12, 24, or 48 h.

### 4.7. Statistical Analysis

Statistical analyses were performed by using GraphPad Prism 9 (Version 9.5.0 GraphPad Software, LLC). The 2^−ΔCt^ values were used for statistical testing, whereas fold-change values were calculated and reported solely for data presentation and biological interpretation. A multiple unpaired t-test was applied to RT-qPCR. Data are shown as mean ± SD of at least three independent biological replicates, where each point represents the median point of three technical replicates acquired for each experiment. Correction for multiple testing was performed by Holm–Šidák correction. Statistical significance was set at *p*-value < 0.05.

## 5. Conclusions

This study highlights that EGFR-mutated cells treated with EGFR-TKIs, with or without TGF-β, show effects on EMT markers after 96 h, namely a reduction of E-cadherin and an increase of N-cadherin, like those observed at 24 h in wild-type EGFR cells treated with TGF-β alone. Instead, a similar effect on vimentin is not observed, which perhaps requires a longer-lasting exposure. This suggests an activation of EMT already in the early phases of treatment with EGFR-TKIs. Furthermore, among EMT-TFs, Slug seems to have a prominent role also with osimertinib, as is already known for gefitinib. The presence of a prevalent TP53 mutation together with the EGFR exon 19 mutation in PC9 cells could prepare the resistance to EGFR-TKIs mediated by Slug overexpression, in accordance with what has already been demonstrated in other studies.

## Data Availability

Raw data are available from the Corresponding Authors through reasonable request.

## Supplementary Materials

The following supporting information can be downloaded at: https://www.mdpi.com/article/10.3390/ijms27146207/s1.

## Abbreviations

The following abbreviations are used in this manuscript:

BRAF	V-raf murine sarcoma viral oncogene homolog B1
cDNA	complementary DNA
CGC	Cancer Gene Census
COSMIC	Catalogue of Somatic Mutations in Cancer
EGFR	Epidermal Growth Factor Receptor
EML4-ALK	Echinoderm microtubule-associated protein-like 4-anaplastic lymphoma kinase
EMT	Epitelial-to-Mesenchymal Transition
HER2	Human Epidermal Growth factor Receptor 2
KRAS	Kirsten rat sarcoma virus
MET	Mesenchymal–epithelial transition factor
MTT	3-(4,5-dimethylthiazol-2-yl)-2,5-diphenyltetrazolium bromide
NGS	Next-Generation Sequencing
NSCLC	Non-Small Cell Lung Cancer
NTRK	Neurotrophic tropomyosin kinase receptor
PI3KCA	Phosphatidylinositol 3-kinase
RET	Rearranged during transfection
ROS1	C-ros proto-oncogene 1
TGF-β	Transforming Growth Factor-β
TKI	Tyrosine Kinase Inhibitors
SCLC	Small-Cell Lung Cancer
VAF	Variant Allele Frequency
