# Supplementary material for: Early-Phase Activation of Epithelial–Mesenchymal Transition in Lung Cancer Cells Treated with Epidermal Growth Factor Receptor Tyrosine Kinase Inhibitors
Source: Int J Mol Sci. 2026 Jul 11;27(14):6207. doi: 10.3390/ijms27146207 (PMC13409766; doi:10.3390/ijms27146207)
Supplement: Supplementary file 1 [file ijms-27-06207-s001.zip › ijms-3899480-supplementary.pdf]

Supplementary Table S1: List of Ct in real-time PCR of the used reference β-actin.

	B-actin Ct
A549 ctr 24h	10,90
A549 ctr 96h	11,07
PC9 ctr 24h	11,05
PC9 ctr 96h	11,06
A549 TGFb 24h	10,26
A549 TGFb 96h	11,26
PC9 TGFb 24h	11,18
PC9 TGFb 96h	11,28
PC9 Gef 24h	10,14
PC9 Gef 96h	10,07
PC9 Osi 24h	10,45
PC9 Osi 96h	10,28
PC9 Gef+TGFb 24h	10,40
PC9 Gef+TGFb 96h	10,05
PC9 Osi+TGFb 24h	10,57
PC9 Osi+TGFb 96h	10,47

Supplementary Table S2. Variant allele frequency of gene mutations in A549 cell line. Variants in coding DNA and protein change are reported, as well as tier of mutation clinical actionability according to The Cancer Gene Census (CGC) within the Catalogue of Somatic Mutations in Cancer (COSMIC).

**Gene**	**Variant**			**Variant allele frequency (%)**			
				**24 hours**		**96 hours**	
	**Variant (coding DNA)**	**Variant (protein)**	**Tier**	**Control 24 h**	**TGF-β 24 h**	**Control 96 h**	**TGF-β 96 h**
KRAS	c.34G>A	G12S	II	100	100	100	100
STK11	c.109C>T	Q37*	II	100	100	100	100
ATR	c.2634-1G>A	N/A	II	35	38	39	40
SMARCA4	c.2184_2206del	Q729C_fs*4	III	100	100	100	100
SMO	c.808G>A	V270I	IV	65	68	66	64
FLT3	c.969C>A	N323K	IV	52	46	52	49

Supplementary Table S3: Variant allele frequency of gene mutations in PC9 cell line. Variants in coding DNA and protein change are reported, as well as tier of mutation actionability, according to The Cancer Gene Census (CGC) within the Catalogue of Somatic Mutations in Cancer (COSMIC). Gef: Gefitinib; Osi: Osimertinib.

Gene	Variant			Variant allele frequency (%)							
				24 hours		96 hours					
	Variant (coding DNA)	Variant (protein)	Tier	Control 24 h	TGF-β 24 h	Control 96 h	TGF-β 96 h	Gefitinib	Osimertinib	Gefitinib +TGF-β	Gefitinib
EGFR	c.2235_2249del	E746_A750del	I	88	89	91	88	93	92	93	89
CREBBP	c.5725C>A	P1909T	III	29	28	28	30	30	34	30	29
NOTCH3	c.5677C>T	R1893*	III	26	27	25	28	23	25	23	25
TP53	c.743G>A	R248Q	III	100	100	100	100	100	100	100	100
SLX4	c.5477G>A	R1826Q	IV	54	55	55	56	54	54	54	56
ATM	c.1526G>C	G509A	IV	30	30	32	31	30	33	30	37

Supplementary Table S4: Multiple t-test for transcription factors expression after 24h vs. 96h of treatment with TGF-β +/- anti-EGFR tyrosine inhibitors in A549 and PC9 cell lines. Reported p-values are relative X-fold values difference between 24h and 96h treatment, as normalized to the relative mean value of the control. *ns*: not significant.

Experimental groups comparison of 24h vs 96h treatment		Transcription factor exact p-value for 24 vs. 96h of treatment				
**Cell Line**	**Treatment**	**Snail**	**Slug**	**Zeb1**	**Zeb2**	**Twist**
A549	TGF-β	*ns*	*ns*	*ns*	*ns*	*ns*
PC9	TGF-β	*ns*	0.013153	*ns*	*ns*	*ns*
PC9	Gefitinib	*ns*	*ns*	*ns*	*ns*	*ns*
PC9	Gefitinib + TGF-β	*ns*	0.001328	*ns*	<0.00005	*ns*
PC9	Osimertinib	*ns*	*ns*	0.007627	0.000018	0.045076
PC9	Osimertinib + TGF-β	*ns*	0.000888	*ns*	*ns*	*ns*
